# Differential Influence of Sample Sex and Neuronal Maturation on mRNA and Protein Transport in Induced Human Neurons

**DOI:** 10.3389/fnmol.2020.00046

**Published:** 2020-04-03

**Authors:** Baojin Ding, Masuma Akter, Chun-Li Zhang

**Affiliations:** ^1^Department of Biology, University of Louisiana at Lafayette, Lafayette, LA, United States; ^2^Department of Molecular Biology, University of Texas Southwestern Medical Center, Dallas, TX, United States; ^3^Hamon Center for Regenerative Science and Medicine, University of Texas Southwestern Medical Center, Dallas, TX, United States

**Keywords:** reprogrammed human neurons, nucleocytoplasmic transport, nuclear mRNA export, fluorescence *in situ* hybridization (FISH), protein nuclear transport, sex as a biological variable (SABV)

## Abstract

Nucleocytoplasmic transport (NCT) across thenuclear envelope (NE) is tightly regulated in eukaryotic cells and iscritical for maintaining cellular homeostasis. Its dysregulationleads to aging and neurodegeneration. Because they maintainaging-associated hallmarks, directly reprogrammed neurons from human fibroblasts are invaluable in understanding NCT. However, it is not clear whether NCT activity is influenced by neuronal maturation and sample sex [a key biological variable emphasized by the National Institutes of Health (NIH) policy]. We examined here NCT activity at the single-cell level by measuring mRNA subcellular distribution and protein transport in directly induced motor neurons (diMNs) from adult human fibroblasts. The results show that mRNA subcellular distribution but not protein transport is affected by neuronal maturation stages, whereas both transport processes are not influenced by the sample sex. This study also provides quantitative methods and optimized conditions for measuring NCTs of mRNAs or protein cargoes, establishing a robust way for future functional examinations of NCT activity in directly induced neurons from diseased human patients.

## Introduction

In eukaryotic cells, transcription and translation processes are physically separated by the nuclear envelope (NE). Newly transcribed mRNAs must be exported to the cytoplasm for protein synthesis, while some proteins require to be imported into the nucleus to fulfill their nuclear functions. The nuclear pore complex (NPC) is the principal gateway between the nucleus and cytoplasm. It is one of the largest protein complexes in eukaryotic cells, penetrating and bridging the inner and outer nuclear membrane (Alber et al., 2007; Mohr et al., 2009). In vertebrates, a fully assembled NPC has an estimated molecular mass of 120 MDa, composed of multiple copies of about 30 different proteins that are called nucleoporins (Nups; Beck and Hurt, 2017). Its three-dimensional structure shows an eight-fold rotational symmetry and consists of several major domains, such as cytoplasmic filaments, nuclear basket, central transport channel, and a core scaffold that supports the central channel (Alber et al., 2007; Kabachinski and Schwartz, 2015). The central channel is filled and surrounded with a distinct class of Nups (Grunwald et al., 2011), which contain phenylalanine and glycine (FG) repeats. FG repeats are intrinsically disordered domains (Lemke, 2016), and they directly function in nucleocytoplasmic transport (NCT) through mediating the passage of the soluble transport receptors (Frey et al., 2006; Frey and Gorlich, 2007; Grunwald et al., 2011).

Generally, cargoes of less than 40–60 kDa can passively diffuse through the NPC, but transport of larger macromolecules through the NPC requires the receptor-mediated transport pathways. Although different kinds of cargoes could be mediated by different transport pathways, a general paradigm usually involves different nuclear transport receptors, the small GTPase Ran and its regulatory factors (Grunwald et al., 2011). Many nuclear transport receptors belong to the karyopherin families, including importins and exportins. Importins recognize nuclear localization sequence (NLS) on their cargo proteins and mediate their import into the nucleus. Exportins recognize nuclear export sequence (NES) and mediate their cargo protein export (Lange et al., 2007; Stewart, 2007a). These karyopherins bind NLSs or NESs of their cargoes to the FG Nups and to the GTPase Ran (Moore and Blobel, 1993). The intrinsic GTPase activity of Ran is low, but interactions with Ran binding proteins (RanBPs) and the Ran-GTPase-activating protein (RanGAP) stimulate GTP hydrolysis. RanBPs are large scaffolding proteins that bind Ran and RanGAP. Because RanBPs are anchored in the cytoplasm side of the nuclear membrane, efficient conversion of RanGTP to RanGDP will occur only in the cytoplasm, yielding a nuclear/cytoplasm ratio of RanGTP of approximately 200:1 (Pollard et al., 2017). On the other hand, another Ran regulatory factor, Ran-GDP-exchange factor (Ran-GEF), switches the RanGDP-bound state into a RanGTP-bound state in the nucleus. This exchange further strengthens the differences of the subcellular distribution: a higher RanGDP concentration in the cytoplasm and a higher RanGTP concentration in the nucleus. This RanGTP-RanGDP gradient across the nuclear membrane generates a driving force for directional NCT processes (Kopito and Elbaum, 2007; Terry and Wente, 2009).

The exportins of karyopherin family and Ran cycle also regulate the export of transfer RNAs (tRNAs), micro RNAs (miRNAs), small nuclear RNAs (snRNAs), and ribosomal RNAs (rRNAs; Rodriguez et al., 2004). However, the export of mRNA is mechanistically different from proteins and other RNAs because it uses a non-karyopherin transport receptor and does not directly depend on the RanGTP–RanGDP gradient. mRNA is exported as a large messenger ribonucleoprotein (mRNP) complex, in which a single mRNA is associated with RNA-binding proteins (RNPs) that have functions in processing, capping, splicing, and polyadenylation (Kabachinski and Schwartz, 2015). In humans, the Nxf1–Nxt1 heterodimer functions as a general mature mRNP export receptor (Stewart, 2007b). Like karyopherins, the Nxf1–Nxt1 complexes can physically interact with FG Nups and mediate RNP cargoes crossing the nuclear membrane through the NPC. However, once part of the mRNP reaches the cytoplasmic face of the NPC, the transport receptor Nxf1–Nxt1 heterodimer will be released in an ATP-dependent manner, rather than by GTP hydrolysis (Montpetit et al., 2011). The protein export receptor Crm1/Xpo1 is also involved in the nuclear export of a number of unspliced and partially spliced viral mRNAs (Forbes et al., 2015). Besides the NPC-dependent pathway for nuclear mRNA export, another NPC-independent mechanism was identified in *Drosophila*. In this mechanism, megaRNP (ultra-large ribonucleoprotein) granules containing mRNAs exit the nucleus *via* budding through the nuclear membranes without using a nuclear pore complex (Speese et al., 2012). This NE-budding pathway is likely utilized in cell processes or stages that require high levels of protein synthesis (Jokhi et al., 2013).

Dysregulations of NCT have been shown to be implicated in aging (Mertens et al., 2015) and neurological diseases, such as amyotrophic lateral sclerosis (ALS; Freibaum et al., 2015; Jovicic et al., 2015; Zhang et al., 2015; Kim and Taylor, 2017; Chou et al., 2018; Burk and Pasterkamp, 2019), Huntington’s disease (HD; Gasset-Rosa et al., 2017; Grima et al., 2017), and Alzheimer’s disease (AD; Eftekharzadeh et al., 2018; Lester and Parker, 2018). Thus, to decipher the pathophysiological mechanisms underlying NCT-defective neurological diseases, the examination of NCT activity in human neurons is extremely critical. Excitingly, direct conversion of neurons from adult fibroblasts overcomes the limited access to human neuronal samples, providing an unprecedented approach in the research of neurological diseases (Mertens et al., 2015; Liu et al., 2016; Victor et al., 2018). How can the NCT activity unbiasedly be examined in these reprogrammed human neurons? Do the reprogramming stages and sample sex affect the NCT activity? The answers to these questions are yet to be elucidated. Particularly, sex as a biological variable (SABV) is a key part of the new National Institutes of Health (NIH) initiative to enhance reproducibility through rigor and transparency (Clayton, 2018). In this study, we attempted to address these questions in motor neurons (MNs) that were directly converted from skin fibroblasts of both male and female healthy human donors. Nuclear mRNA export is measured by fluorescence *in situ* hybridization (FISH) with digoxigenin (DIG)-labeled oligo-dT probes, and protein NCT is examined by a dual reporter system. These approaches gave rise to strong and reliable results. We also found that developmental stages, but not sample sex, significantly affect the mRNA subcellular distribution. These results provide quantitative methods in the measurement of nuclear transport of both mRNA and protein cargoes, paving the way for further examination of NCT activity in reprogrammed patient-specific neurons.

## Materials and Methods

### Cell Lines and Culture Conditions

Human embryonic kidney (HEK) 293T cells [American Type Culture Collection (ATCC)] were grown in Dulbecco’s modified Eagle’s medium (DMEM; Gibco) containing 10% heat-inactivated fetal bovine serum (FBS, Gibco) and 1% penicillin/streptomycin (Gibco). Primary skin fibroblasts from apparently healthy human individuals were obtained from the cell repository at the Coriell Institute for Medical Research (Table 1). They were cultured in DMEM supplemented with 15% FBS and 1% penicillin/streptomycin. Two different cell culture media were sequentially employed in reprogramming human neurons. (1) Neuronal induction medium consists of DMEM: F12: neurobasal (1:1:1), 0.8% N2 (Invitrogen), 0.8% B27 (Invitrogen), 1% penicillin/streptomycin, and supplemented with 10 μM forskolin (FSK; Sigma-Adrich, St. Louis, MO, USA), 1 μM dorsomorphin (DM; Millipore, Kankakee, IL, USA), and 10 ng/ml basic fibroblast growth factor (bFGF; PeproTech, Rocky Hill, NJ, USA). (2) Neuronal maturation medium consists of DMEM: F12: neurobasal (1:1:1), 0.8% N2, 0.8% B27, 1% penicillin/streptomycin, and supplemented with 5 μM FSK and 10 ng/ml each of brain-derived neurotrophic factor (BDNF; PeproTech, Rocky Hill, NJ, USA), glial cell-derived neurotrophic factor (GDNF; PeproTech, Rocky Hill, NJ, USA), and neurotrophin-3 (NT3; PeproTech, Rocky Hill, NJ, USA).

**Table 1:** Fibroblast cell lines used in this study.

	Coriell Cat#	RRID	Sex	Age at sampling	Tissue type	Product
F1	GM07522	CVCL_F162	Female	19 years	Skin	Fibroblast
F2	GM04506	CVCL_7413	Female	20 years	Skin	Fibroblast
F3	GM08400	CVCL_7483	Female	37 years	Skin	Fibroblast
M1	GM03652	CVCL_7397	Male	24 years	Skin	Fibroblast
M2	GM00495	CVCL_7289	Male	29 years	Skin	Fibroblast
M3	GM00024	CVCL_7269	Male	31 years	Skin	Fibroblast

*RRID, Research Resource Identifier*.

### Plasmids and Virus Production

As previously descried (Liu et al., 2016), a third-generation lentiviral vector (pCSC-SP-PW-IRES-GFP) was used to express NEUROG2-IRES-GFP-T2A-Sox11 and ISL1-T2A-LHX3. The dual reporter 2Gi2R was generously provided by Dr. Fred H. Gage (Salk Institute for Biological Studies; Mertens et al., 2015). Replication-incompetent lentiviruses were produced in HEK293T cells, and viral supernatants were collected at 48 and 72 h post-transfection. Viral supernatants were filtered through 0.45-μm syringe filters and titered as previously described (Ding and Kilpatrick, 2013). Lentiviruses were stored at 4°C prior to cell transduction.

### Direct Conversion of Human Motor Neurons From Adult Fibroblasts

Lentiviral delivery of four factors (NGN2, Sox11, ISL1, and LHX3) was used as previously described (Liu et al., 2016; Tang et al., 2017). In brief, fibroblasts were plated at a density of 1 × 10^4^ cells/cm^2^ onto Matrigel-coated dishes. Cells were transduced the next day at an approximate multiplicity of infection (MOI) of 10 with lentiviral supernatants supplemented with 6 μg/ml polybrene (Millipore, Kankakee, IL, USA). Fibroblast medium was refreshed after overnight incubation. One day later, the culture was replaced with neuronal induction medium and half-changed every other day until 14 days post infection (dpi). Induced neurons were then partially enriched with a replating procedure (Liu et al., 2013) and cocultured with primary astrocytes on Matrigel-coated coverslips. The medium was half-changed twice a week until analysis. The neuronal conversion efficiency was calculated with the percentage of GFP (+) cells expressing TUBB3 (TUBB3/GFP), and the MN fraction was calculated with the percentage of TUBB3 (+) cells expressing HB9 (HB9/TUBB3).

### Primary Cultures

Primary cortical neurons (PCNs) and cerebellar granule neurons (CGNs) were isolated from CD1 embryonic day-18 mice (E18) and 6-day-old pups (P6) of either sex, respectively, as previously described (Ding et al., 2013, 2016, 2018). All animal work was carried out with permission from the Institutional Animal Care and Use Committee (IACUC) of the University of Texas Southwestern Medical Center. Individual culture experiments were performed using cells prepared from the same litter. Cells were plated at a density of 5 × 10^4^ cells/cm^2^ onto coverslips (Thermo Fisher Scientific, Waltham, MA, USA) in cell culture plates coated with poly-D-lysine/laminin (Invitrogen) in Neurobasal medium (Invitrogen) containing B-27 serum-free supplement (50×; Invitrogen). The medium was half-changed twice a week until analysis.

### Immunocytochemistry

Cells were cultured to the desired time points. They were fixed with 4% paraformaldehyde (PFA; Sigma-Adrich, St. Louis, MO, USA) in phosphate buffered saline (PBS) for 15 min at room temperature (RT) and permeabilized with blocking buffer [PBS containing 3% bovine serum albumin (BSA) and 0.2% Triton X-100] for 1 h. Cells were then incubated with primary antibodies in blocking buffer overnight at 4°C, followed by washing and incubation with fluorophore-conjugated corresponding secondary antibodies. Primary antibodies used in this study are goat anti-CHAT (Millipore, Kankakee, IL, USA; 1:200), mouse anti-HB9 (DSHB; 1:500), chicken anti-MAP2 (Abcam; 1:10,000), mouse or rabbit anti-TUBB3 (Covance; 1:2,000). Nuclei were stained with Hoechst 33342 (HST; Thermo Fisher Scientific, Waltham, MA, USA).

### Fluorescent *in situ* Hybridization

FISH was performed as described previously (Li et al., 2016) with modifications. Briefly, cultured neurons were washed once with DPBS (Gibco) and fixed with 4% PFA in PBS for 30 min at RT. Cells were further permeabilized with 0.2% Triton X-100 (Sigma-Adrich, St. Louis, MO, USA) for 10 min at RT and twice washed with ice-cold PBS. For RNase A-treated controls, samples were incubated with 0.5 mg/ml of RNase A (New England BioLabs) in 10 mM Tris-HCl buffer (pH 7.4) for 1 h at 37°C. The samples were equilibrated with hybridization buffer composed of 2× saline sodium citrate (SSC), 10% dextran sulfate (average molecular weight 500,000; Sigma-Adrich, St. Louis, MO, USA), 10 mM ribonucleoside–vanadyl complex (RVC; New England Biolabs), and 20% formamide (Thermo Fisher Scientific, Waltham, MA, USA). A mixture of *DIG*-labeled oligo-dT or dA probes (0.2 ng/μl) containing yeast tRNA (0.2 μg/μl) were heated to 95°C for 5 min and chilled on ice immediately. Probes were then combined with equal volumes of 2× hybridization buffer and added to samples for overnight incubation at 37°C. Anti-*DIG* antibody (sheep anti-DIG; Sigma-Adrich, St. Louis, MO, USA) and fluorophore-conjugated corresponding secondary antibodies (Thermo Fisher Scientific, Waltham, MA, USA) were used to detect oligo-dT signals. Anti-MAP2 antibody (Abcam) and HST were used to determine neuronal soma and nucleus, respectively. All reagents and solutions were prepared in nuclease-free water.

### Protein Nuclear Transport Assay

Protein nuclear transport was analyzed by using a dual reporter 2Gi2R (Mertens et al., 2015). Cultured cells were transduced with a lentivirus, which expressed a fused double-GFP with an NES (2GFP-NES) and a double RFP containing an NLS (2RFP-NLS). To validate this reporter system, transduced HEK cells were treated with 50 nM leptomycin B (Alfa Aesar) and then analyzed by confocal microscopy during a time course. For directly reprogrammed neurons, the reporter lentivirus was cotransduced with reprogramming factors, and the signal densities and distributions of GFP and RFP were analyzed at the indicated time points.

### Confocal Microscopy and Quantification

Confocal images were obtained with a Zeiss-LSM700 or Leica SP5 confocal microscopes. The ImageJ software (NIH) was used to quantify fluorescence intensity from images of confocal slices. For each neuron, as large an area as possible was measured within the nucleus or cytoplasm. An unbiased approach for data collection was employed. The person who analyzed the images was completely blinded to the sample information. The mean values were used to quantify an average signal density. For FISH assays, the average of oligo-dT signals in soma (both nucleus and cytoplasm) was used to evaluate the overall mRNA expression levels. mRNA subcellular distribution was measured by the ratio of nuclear to cytoplasmic oligo-dT signals (dT_nuc_/dT_cyt_). For the dual reporter assay, GFP and RFP signals were separately measured in the nucleus and cytoplasm. The ratio of nuclear to cytoplasmic GFP (GFPnuc/GFPcyt) was used to evaluate protein export, while the ratio of cytoplasmic to nuclear RFP (RFPcyt/RFPnuc) was used to measure protein nuclear import.

### Statistics

An unpaired two-tailed Student’s *t-*test was used for experiments consisting of two groups. For experiments consisting of more than two groups, one-way analysis of variance (ANOVA) was used with either Tukey (when comparing samples with each other) or Dunnett (when comparing samples to a control) *post hoc* tests. The results are expressed as mean ± SD of three biological replicates, and *P* < 0.05 was treated as significant.

## Results

### Direct Conversation of Adult Human Fibroblasts to Motor Neurons

**Figure 1:**
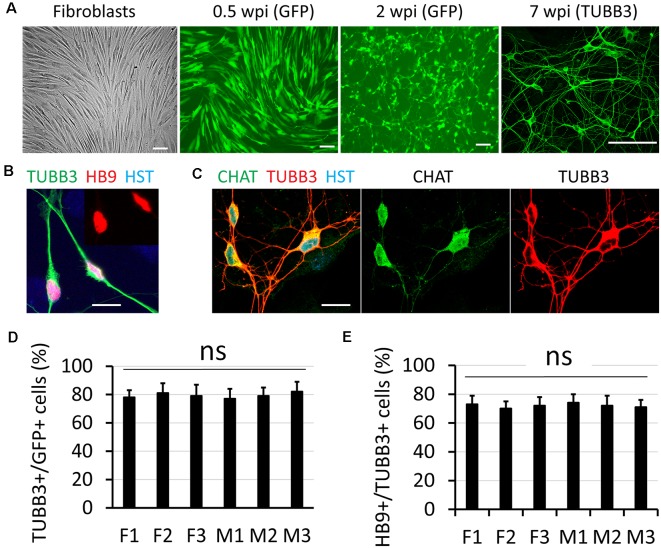
Direct conversation of adult human fibroblasts to motor neurons (MNs). **(A)** Micrographs of fibroblast and directly induced motor neurons (diMNs) at indicated weeks post viral infection (wpi). Transduced cells were identified with co-expressed green fluorescent protein (GFP) at early time points (0.5 and 2 wpi). Successfully converted neurons were identified by neuronal marker of tubulin beta 3 class III (TUBB3). Scale bars: 100 μm. **(B)** Confocal micrograph shows the highly expressed nuclear HB9 (motor neuron and pancreas homeobox 1, the early MN marker) at 2 wpi. The inset shows the nuclear HB9 alone. DNA dye Hoechst 33342 (HST) stained nuclei. Scale bar: 20 μm. **(C)** Confocal micrographs show the robust expression of the mature MN marker choline acetyltransferase (CHAT) at 8 wpi. Scale bar: 20 μm. **(D)** Quantification of the reprogramming efficiency was shown as the percentage of GFP (+) cells expressing TUBB3. More than 300 cells of each line were counted from triplicates. ns, not significant. **(E)** Fractions of diMNs among the reprogrammed neurons were shown as the percentage of TUBB3 (+) cells expressing HB9. More than 300 cells of each line were counted from triplicates. ns, not significant.

Disruption of NCT is particularly involved in movement disorders (Da Cruz and Cleveland, 2016; Li and Lagier-Tourenne, 2018), such as ALS which is also characterized with progressive loss of MNs (Freibaum et al., 2015; Jovicic et al., 2015; Zhang et al., 2015; Boeynaems et al., 2016; Chou et al., 2018; Burk and Pasterkamp, 2019). To understand how defective NCT contributes to diseases, measuring NCT activity directly in human MNs became extremely important. In this study, we generated human MNs from adult fibroblasts through direct reprogramming (Liu et al., 2016). Transduced fibroblasts can be identified with co-expressed GFP, and they gradually became neuron-like and grow complex neurites over time (Figure 1A). Immunocytochemistry confirmed their expression of the neuronal marker tubulin beta 3 class III (TUBB3) and early MN marker HB9 (Figure 1B; Arber et al., 1999). At later stages, such as 6–8 weeks post viral infection (wpi), reprogrammed neurons expressed high levels of choline acetyltransferase (CHAT), the mature MN marker (Figure 1C). Thus, adult fibroblasts could be efficiently and directly converted into MNs, which were hereafter referred as directly induced MNs (diMNs). We examined skin fibroblasts from six apparently healthy donors, three males and three females (Table 1). No significant difference was observed between male and female donors at the neuronal conversion efficiency and the MN fraction (Figures 1D,E). About 80% transduced GFP+ cells expressed TUBB3 (Figure 1D). Among these TUBB3+ neurons, approximately 75% of them highly expressed nuclear HB9 (Figures 1B,E).

Based on this study and previous reports (Mertens et al., 2015; Liu et al., 2016; Smith et al., 2016), we summarized the major features associated with the reprogramming process in Table 2. During the first week, the transduced cells highly express the reprogramming factors, which remodel the chromatin structure and induce global changes on gene expression (Smith et al., 2016). The cells rapidly change morphology with outgrowing neurites and high expression of markers for general neurons at 2–4 wpi (Mertens et al., 2015; Liu et al., 2016). By 6–8 wpi, the reprogrammed neurons become functionally mature, characterized by synaptogenesis, firing of repetitive action potentials, and formation of neuromuscular junctions (NMJs; Liu et al., 2016).

**Table 2:** Biological features associated with the direct reprogramming process.

Stages (wpi)	Major biological features	References
1	High expression of the reprogramming factors and reporter; chromatin remodeling; changes of global gene expression	This study and Smith et al. (2016)
2	Morphological changes with extensive neurite outgrowths; expression of neuronal markers such as TUBB3 and nuclear HB9 in diMNs	This study and Liu et al. (2016)
3	Expression of genes essential for neuronal function: voltage-gated channels, neurotransmitters, transporters, pre- and post-synaptic proteins, etc.; high levels of mRNAs in the nucleus	This study and Mertens et al. (2015)
4	Complex multipolar morphology; robust expression of markers for mature neurons including MAP2 and CHAT; high nuclear mRNA levels	This study and Liu et al. (2016)
6	Typical neuronal morphology; high levels of markers for mature neurons such as CHAT, VAChT, and synaptic proteins; reduced nuclear mRNA levels	This study and Liu et al. (2016)
8	Fully functional neurons with synaptogenesis and mature electrophysiology; formation of neuromuscular junctions between diMNs and myotubes	Liu et al. (2016)

*TUBB3, tubulin beta 3 class III; MAP2, microtubule-associated protein 2; CHAT, choline acetyltransferase; VAChT, vesicular acetylcholine transporter*.

### Measurement of mRNA Distribution in Directly Induced Motor Neurons by Fluorescence *in situ* Hybridization

**Figure 2:**
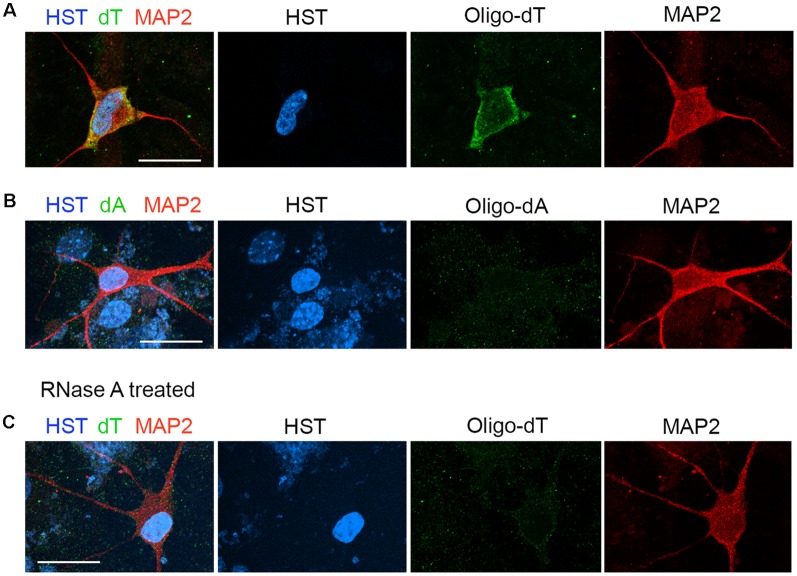
Measurement of the subcellular distribution of mRNA in directly induced motor neurons (diMNs) by fluorescence *in situ* hybridization (FISH). **(A)** FISH analysis of diMNs at 6 weeks post viral infection (wpi) with oligo-dT probes. Microtubule-associated protein 2 (MAP2) was used to define the soma and Hoechst 33342 (HST)-stained nuclei. **(B)** Oligo-dA probes were used as negative controls for FISH assay of diMNs at 6 wpi. **(C)** RNase A-treated diMNs at 6 wpi as negative controls for FISH assay. **(A–C)** Scale bars: 20 μm.

The distribution of total poly(A) RNAs in single diMNs was measured by FISH with oligo-dT probes as previously described (Packard et al., 2015; Li et al., 2016). Following hybridization, immunostaining of microtubule-associated protein 2 (MAP2) was used to identify the soma, and the DNA dye HST was used to define the nucleus. At the late development stage of 6 wpi diMNs, strong oligo-dT signals could be detected in both the nucleus and cytoplasm, but the majority of signals localize in the cytoplasm (Figure 2A). No obvious signals were detected with the oligo-dA probe (Figure 2B) or in diMNs that were treated with RNase A (Figure 2C), suggesting that the oligo-dT signals in FISH assays are specific.

### Neuronal Maturation But Not Sample Sex Affects mRNA Subcellular Distribution in Directly Induced Motor Neurons

**Figure 3:**
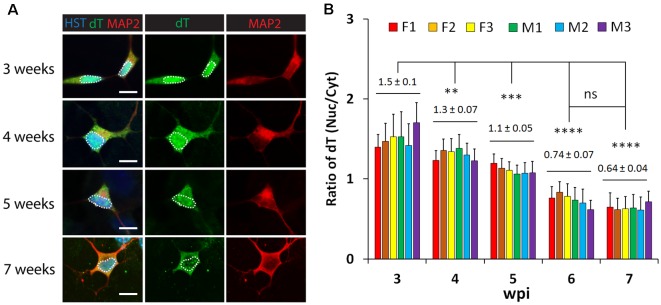
Neuronal maturation but not sample sex affects mRNA subcellular distribution in directly induced motor neurons (diMNs). **(A)** Representative confocal images of FISH analysis of diMNs from 3 to 7 weeks post viral infection (wpi). Microtubule-associated protein 2 (MAP2) was used to define the soma and Hoechst 33342 (HST)-stained nuclei. Nuclei are highlighted in circular dotted lines. Scale bars: 10 μm. **(B)** Quantification of oligo-dT signal distribution in diMNs of each individual at indicated time points. Ratios of the nuclear-to-cytoplasmic dT signal density (Nuc/Cyt) was used to show the mRNA distribution. The labeled average values (mean ± SD) at each time point were calculated from averages of individuals and used for statistical analysis. F, female; M, male. *n*_(neurons)_ > 50 in each sample from triplicates. ns, not significant; **p < 0.01; ***p < 0.001; ****p < 0.0001.

To examine whether neuronal maturation affects mRNA subcellular distribution, we conducted a time-course analysis of diMNs by FISH. At 3 wpi, oligo-dT signals were highly enriched in the nucleus (Figure 3A). Although diMNs at this stage outgrow neurites and express markers for general neurons (MAP2 and TUBB3) and MNs (HB9; Figures 1, Figure 3), their somas are still under transformation from an oval morphology of fibroblasts to the multipolar morphology of typical MNs. With diMN maturation, subcellular distribution of mRNAs gradually changed, with decreased signals in the nucleus and conversely increased signals in the cytoplasm. By 7 wpi, the signal density was obviously higher in the cytoplasm than that in the nucleus (Figure 3A). To quantify the subcellular distribution of poly (A) RNAs, the average signal density was separately measured in the nucleus and cytoplasm. The ratio of nuclear to cytoplasmic oligo-dT signal density (Nuc/Cyt) was significantly decreased within the first 6 wpi but reached a steady state thereafter (Figure 3B). These findings indicate that mRNA subcellular distribution is significantly affected by neuronal maturation stages and that it can be steadily measured at 6 wpi or later. A thorough analysis, however, failed to show any effect of sample sexes on mRNA subcellular localization in diMNs (Figure 3B).

### mRNA Distribution Is Influenced by Neuronal Maturation in Primary Mouse Neurons

**Figure 4:**
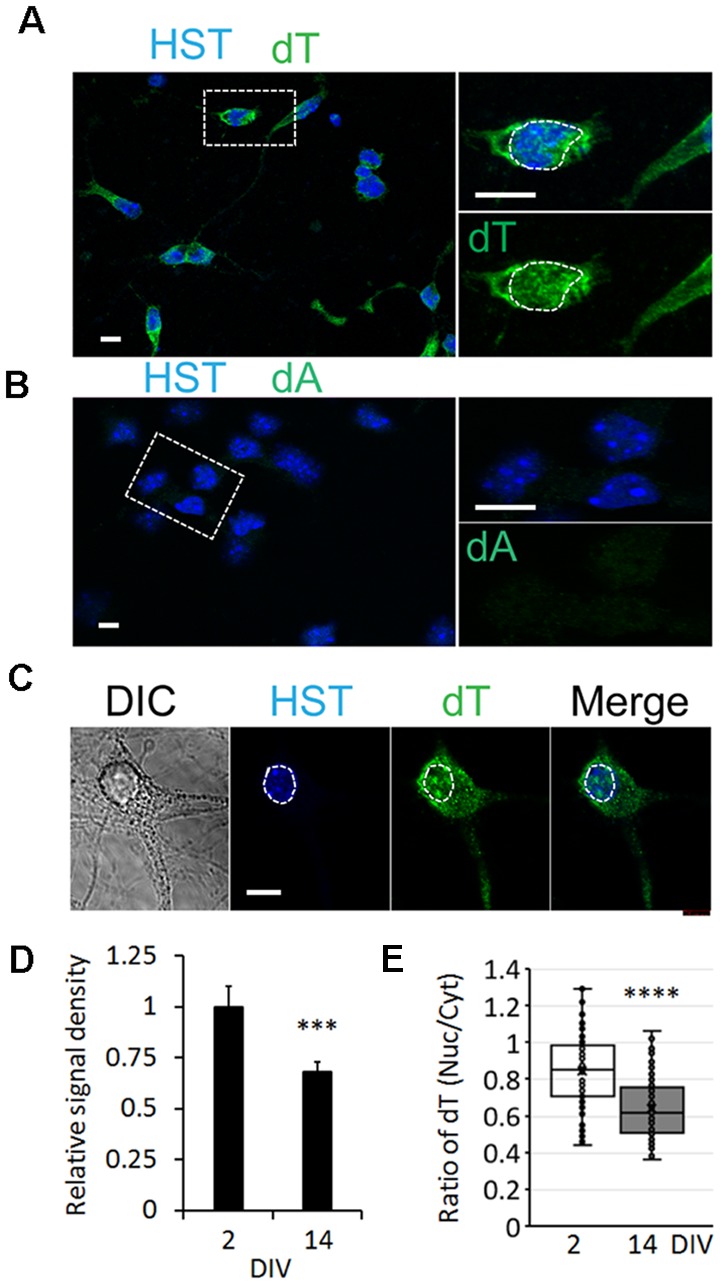
mRNA distribution is influenced by neuronal maturation in primary cortical neurons (PCNs). **(A)** Confocal micrographs of mouse PCNs at 2 days *in vitro* (DIV) with fluorescence *in situ* hybridization (FISH) analysis. The highlighted area with dotted rectangle is also shown in a large magnification on the right. Hoechst 33342 (HST)-stained nuclei. Scale bar: 10 μm. **(B)** Oligo-dA probes were used as negative control for FISH assay in mouse PCNs at 2 DIV. The highlighted area with dotted rectangle is also shown in a large magnification on the right. Scale bar: 10 μm. **(C)** FISH analysis of mouse PCNs at 14 DIV. The nucleus is highlighted in a circular dotted line. DIC, differential interference contrast. Scale bar: 10 μm. **(D)** Relative cellular oligo-dT signal density in PCNs at 2 and 14 DIV. The value of 2 DIV was set as 1. ***p < 0.001. *n*_(neurons)_ = 86 for 2 DIV and *n* = 78 for 14 DIV from triplicates. **(E)** Quantification of oligo-dT signal distribution in PCNs at 2 and 14 DIV. ****p < 0.0001. *n*_(neurons)_ = 91 for 2 DIV and *n* = 87 for 14 DIV from triplicates.

**Figure 5:**
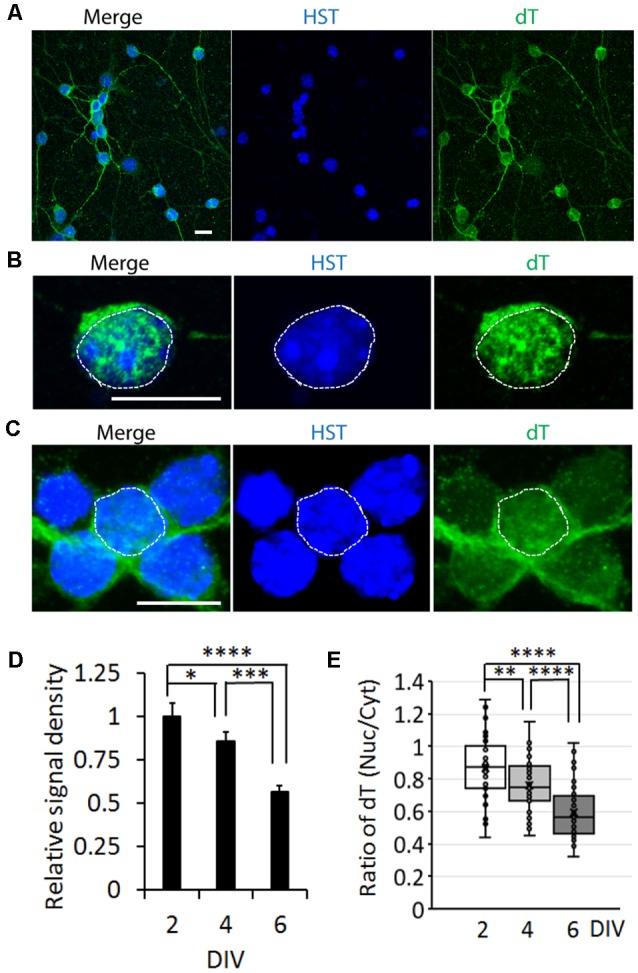
mRNA distribution is influenced by neuronal maturation in cerebellar granule neurons (CGNs). **(A)** Low magnification of confocal micrographs of mouse primary CGNs at 4 days *in vitro* (DIV) with fluorescence *in situ* hybridization (FISH) analysis. Hoechst 33342 (HST)-stained nuclei. Scale bar: 10 μm. **(B)** A representative confocal micrograph of a CGN at 2 DIV with FISH analysis. The nucleus is highlighted with circular dotted line based on the HST signal. Scale bar: 10 μm. **(C)** FISH analysis of CGNs at 6 DIV. One nucleus is highlighted with circular dotted line based on the HST signal. Scale bar: 10 μm. **(D)** Relative cellular oligo-dT signal density in CGNs at 2, 4, and 6 DIV. The value of 2 DIV was set as 1. *n*_(neurons)_ = 76 for 2 DIV, *n* = 72 for 4 DIV and *n* = 65 for 6 DIV from triplicates. *p < 0.01, ***p < 0.001; ****p < 0.0001. **(E)** Quantification of oligo-dT signal distribution in CGNs at 2, 4, and 6 DIV. *n*_(neurons)_ = 71 for 2 DIV, *n* = 65 for 4 DIV, and *n* = 64 for 6 DIV from triplicates. **p < 0.01; ****p < 0.0001.

To determine whether the influence of neuronal maturation on mRNA subcellular distribution is a common feature or unique to diMNs, we examined primary mouse neurons at different maturation stages. Primary mouse cortical neurons (PCNs) were isolated from E18 embryos. After culturing for 2 days *in vitro* (DIV), they showed strong oligo-dT signals in both the nucleus and the cytoplasm (Figure 4A). The specificity of FISH assays was verified by using the oligo-dA probe that showed no any signals (Figure 4B). The overall oligo-dT signals and the ratio of nuclear to cytoplasmic signal density (Nuc/Cyt) significantly decreased when examined at 14 DIV (Figures 4C–E). We also analyzed mRNA distribution in primary mouse CGNs, which were isolated from P6 pups (Figures 5A–C). These postnatal neurons mature faster than those prenatal PCNs in culture, and they are considered as mature with robust marker expression at 6 DIV (Ding et al., 2013; Leto et al., 2016). Consistent with the results in PCNs, total mRNA levels and the ratio of nuclear to cytoplasmic signal density (Nuc/Cyt) gradually decreased with the CGN maturation process (Figures 5D,E). These results clearly indicate that the influence of neuronal maturation on mRNA subcellular distribution is not unique to diMNs but also occurs in primary mouse neurons.

### Protein Nucleocytoplasmic Transport Measurement

**Figure 6:**
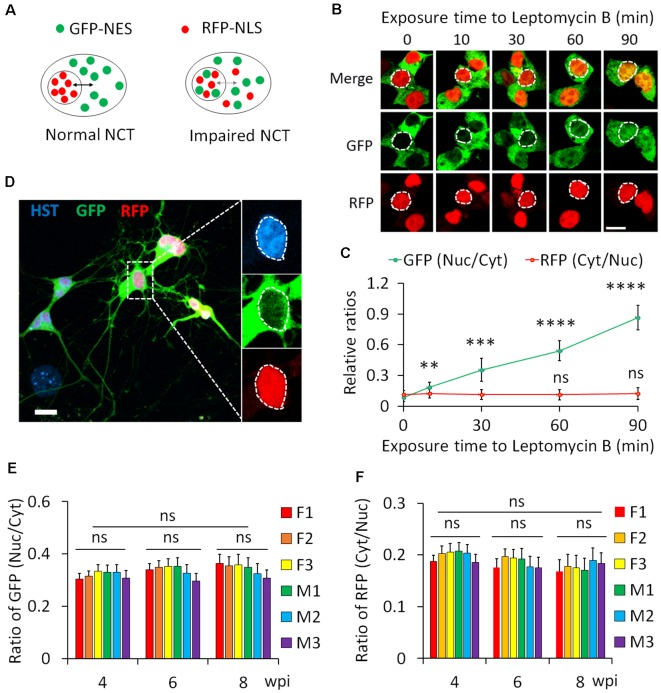
Protein nucleocytoplasmic transport (NCT) measurement with a dual reporter. **(A)** A schematic shows a dual reporter system for measuring protein NCT. GFP, green fluorescent protein; NES, nuclear export sequence; RFP, red fluorescent protein; NLS, nuclear localization sequence. **(B)** Representative confocal micrographs of human embryonic kidney (HEK) cells expressing the dual reporter with and without leptomycin B (50 nM) treatment. Circular dotted lines highlight the nuclei. Scale bar: 10 μm. **(C)** Ratios of nuclear to cytoplasmic GFP-NES (Nuc/Cyt) and cytoplasmic to nuclear RFP-NLS (Cyt/Nuc) in HEK cells. *n*_(cells)_ > 100 for each time point from triplicates. Compare to control (0 min), ns, not significant; **p < 0.01, ***p < 0.001; ****p < 0.0001. **(D)** A representative confocal micrograph of directly induced motor neurons (diMNs) expressing the dual reporter at 6 weeks post viral infection (wpi). The highlighted area was also shown at a large magnification, and the nucleus is highlighted by a circular dotted line based on the Hoechst 33342 (HST) signal. Scale bar: 10 μm. **(E)** Ratio of nuclear to cytoplasmic GFP-NES (Nuc/Cyt) in diMNs at 4, 6, and 8 wpi from each donor. The average values of each time point were calculated from averages of individuals and used for statistical analysis. F, female; M, male. *n*_(neurons)_ < 50 for each sample from triplicates. ns, not significant. **(F)** Ratio of cytoplasmic to nuclear RFP-NLS (Cyt/Nuc) in diMNs at 4, 6, and 8 wpi from each donor. The average values of each time point were calculated from averages of individuals and used for statistical analysis. F, female; M, male. *n*_(neurons)_ < 50 for each sample from triplicates. ns, not significant.

We employed a dual reporter system (2Gi2R) to measure protein NCT activity in single diMNs. This reporter system consists of a fused double-GFP with an NES (2GFP-NES) and a double RFP containing an NLS (2RFP-NLS; Mertens et al., 2015). NES and NLS are conserved signal sequences that are recognized by nuclear transport receptors of exportins and importins, respectively (Christie et al., 2016; Cagatay and Chook, 2018). Thus, both protein export and import can be analyzed with this dual reporter in single cells. In cells with normal NCT activity, GFP and RFP are respectively localized in the cytoplasm and nucleus, whereas such distribution will be disrupted when NCT activity is impaired (Figure 6A). An increased ratio of nuclear to cytoplasmic GFP (GFP_nuc_/GFP_cyt_) represents disrupted protein export, while a higher ratio of cytoplasmic to nuclear RFP (RFP_cyt_/RFP_nuc_) indicates a compromised protein nuclear import.

We first validated the 2Gi2R reporter in HEK cells with the treatment of leptomycin B, a nuclear export inhibitor (Jang et al., 2003). Both GFP and RFP were exclusively localized in the cytoplasm and nucleus of transduced cells, respectively (Figure 6B). In the presence of leptomycin B, GFP gradually accumulated inside the nucleus. A significant increase of the ratio of nuclear to cytoplasmic GFP (GFP_nuc_/GFP_cyt_) was observed at 10 min and later following exposure to leptomycin B (Figures 6B,C). On the other hand, the distribution of RFP was not significantly affected (Figures 6B,C). These results agree well with previous reports that leptomycin B is a highly specific inhibitor of nuclear export receptor CRM1 (Jang et al., 2003; Mutka et al., 2009), suggesting that this reporter system is reliable in protein NCT measurement.

Human fibroblasts from healthy donors were then transduced with lentiviruses expressing the 2Gi2R reporter and the reprogramming factors. In the resulting diMNs, both GFP and RFP were mainly localized in the cytoplasm and nucleus, respectively (Figure 6D). We also examined but failed to observe an effect of neuronal maturation on protein NCT activity, indicated by unaltered subcellular distribution of the GFP and RFP reporters at 4, 6, or 8 wpi (Figures 6E,F). Similarly, protein NCT activity is not affected by sample sexes during this time-course analysis (Figures 6E,F).

## Discussion

In this study, we examined nuclear transport of both mRNAs and proteins in diMNs from adult human fibroblasts. Combining confocal microscopy with quantitative single-cell analysis, the results of this study demonstrate a robust way to measure NCT of both mRNA and protein cargoes in cultured neurons. Importantly, a time-course analysis revealed that neuronal maturation but not sample sex significantly affects mRNA subcellular distribution, whereas both of these biological variables have no effect on protein transport. These results pave a way for future detailed investigations of NCT activity under pathological conditions.

Defective NCT has emerged as a common pathogenic factor of many neurodegenerative diseases, including the movement disorder ALS (Freibaum et al., 2015; Jovicic et al., 2015; Zhang et al., 2015; Chou et al., 2018; Burk and Pasterkamp, 2019). To understand the contributions of disrupted NCT to the pathogenesis of diseases, examinations of NCT activity directly in reprogrammed human neurons became invaluable approaches. Human neurons could be generated by direct conversion from patient fibroblasts or induced pluripotent stem cells (iPSCs)-based reprogramming and differentiation (Barker et al., 2018; Mertens et al., 2018). As iPSCs are epigenetically reset to an embryonic state during the reprogramming process, certain age-associated features are erased in iPSC-neurons (Lapasset et al., 2011; Soria-Valles and López-Otín, 2016). Directly reprogrammed neurons from donors can maintain aging-associated hallmarks, providing an excellent model system in the research of age-related neurodegenerative diseases (Mertens et al., 2015, 2018; Tang et al., 2017; Victor et al., 2018).

In this study, using diMNs, we have systematically examined the nuclear mRNA export by FISH with digoxigenin-labeled oligo-dT probes. Compared to directly fluorophore-conjugated probes, the oligo-dT signals in this method could be further amplified by anti-digoxigenin antibody and fluorophore-conjugated secondary antibodies, providing much stronger signals for the quantification assay. Most importantly, the specificity of oligo-dT signals was confirmed by a lack of signal when digoxigenin-labeled oligo-dA was used as probes, or when samples were treated with RNase A. Combined with immunostaining of neuron markers and nuclear dye, the soma and the nucleus of neurons can be clearly defined. Thus, the nuclear and cytoplasmic mRNAs can be measured, and their ratio will reveal the subcellular distribution of mRNAs. The increased ratio of nuclear to cytoplasmic oligo-dT signals (dT_nuc_/dT_cyt_) will indicate the impaired nuclear mRNA export. Time-course analysis indicated that neuronal maturation significantly affected the mRNA subcellular distribution in reprogrammed MNs. Interestingly, similar effects were observed when examining developmental stages on the mRNA subcellular distribution in mouse primary neurons. This could be due to the higher nuclear transcriptional levels at early developmental stages when neurons undergo rapid neurite outgrowth. Thus, to obtain reliable results of NCT activity, using reprogrammed human neurons at late mature stages is vitally critical. In our condition, the best developmental stage to measure the mRNA subcellular distribution of diMNs is 6 wpi.

Compared to nuclear mRNA export, different signaling pathways are employed for protein nuclear transport (Stewart, 2007a; Kabachinski and Schwartz, 2015). In this study, we also simultaneously analyzed protein nuclear import and export using a dual reporter, in which GFP-NES and RFP-NLS were co-expressed in transduced neurons. By 4 wpi, both GFP and RFP signals are strong enough for quantification analysis. The increased ratio of nuclear to cytoplasmic GFP (GFP_nuc_/GFP_cyt_) will suggest the impaired protein export, while the higher ratio of cytoplasmic to nuclear RFP (RFP_cyt_/RFP_nuc_) will indicate the compromised protein nuclear import. Consistent with the results of mRNA subcellular distribution in diMNs, the sample sex did not significantly affect protein nuclear transport. Different from mRNA subcellular distribution, protein NCT did not show any significant difference in diMNs within 4–8 wpi, suggesting that protein NCT activity does not significantly change within this time frame. The differential influence of reprogramming stages on mRNA and protein NCT could be because they are mediated by different signaling pathways. Our results also support the notion that the higher transcriptional levels in early-stage neurons, rather than the NCT machinery itself, significantly influence the poly (A) RNA subcellular distribution.

However, we did not analyze the protein NCT at time points earlier than 4 wpi because of the relatively low expression levels of both GFP-NES and RFP-NLS. Therefore, the differences of protein NCT activity at very early reprogramming stages cannot be excluded as a possibility. Another limitation in this study is that only overall poly (A) RNAs have been examined, rather than the specific transcripts. If digoxigenin-labeled gene-specific probes are employed, we are confident that the subcellular distribution of a particular mRNA can be measured in cultured neurons using our approach. The measurement of the subcellular distribution of specific transcripts would be more important in identification of mislocalized mRNAs in patient-derived neurons. Our study indicates that the sample sex does not significantly influence the mRNA subcellular distribution and protein NCT in diMNs. However, given the facts that sample sex is a biological variable in many biological processes, sex-matched controls are still recommended in using reprogrammed human neurons to model human diseases.

## Data Availability Statement

All datasets generated for this study are included in the article.

## Ethics Statement

All animal work was carried out with approvals from the Institutional Animal Care and Use Committee (IACUC) at the University of Texas Southwestern Medical Center. Primary human skin fibroblasts were all obtained from the cell repository at the Coriell Institute for Medical Research. No approval was required from the institutional review board (IRB) at University of Texas Southwestern Medical Center.

## Author Contributions

BD conceived and performed most experiments and acquired and analyzed data. MA performed leptomycin B experiment. BD and C-LZ prepared the manuscript. All authors reviewed and approved the final manuscript.

## Conflict of Interest

The authors declare that the research was conducted in the absence of any commercial or financial relationships that could be construed as a potential conflict of interest.

## Abbreviations

AD: Alzheimer’s disease
ALS: amyotrophic lateral sclerosis
BDNF: brain-derived neurotrophic factor
CGN: cerebellar granule neuron
CHAT: choline acetyltransferase
DIG: digoxigenin
DIV: days *in vitro*
DM: dorsomorphin
dpi: days post infection
FBS: fetal bovine serum
FGF: fibroblast growth factor
bFGF: basic fibroblast growth factor
FISH: fluorescence *in situ* hybridization
FSK: forskolin
GDNF: glial cell-derived neurotrophic factor
GFP: green fluorescent protein
HEK: human embryonic kidney
HST: Hoechst 33342
HD: Huntington’s disease
MAP2: microtubule-associated protein 2
MN: motor neuron
diMN: directly induced motor neuron
MOI: multiplicity of infection
NCT: nucleocytoplasmic transport
NE: nuclear envelope
NES: nuclear export sequence
NLS: nuclear localization sequence
NPC: nuclear pore complex
NT3: neurotrophin-3
Nups: nucleoporins
PCN: primary cortical neuron
iPSC: induced pluripotent stem cell
RanBP: Ran binding protein
RanGAP: Ran-GTPase-activating protein
Ran-GEF: Ran-GDP-exchange factor
RFP: red fluorescent protein
RNP: ribonucleoprotein
mRNP: messenger ribonucleoprotein
megaRNP: ultra-large ribonucleoprotein
RVC: ribonucleoside–vanadyl complex
SSC: saline sodium citrate
TUBB3: tubulin beta 3 class III
wpi: weeks post viral infection.
